# Cholesterol-conjugated poly(D, L-lactide)-based micelles as a nanocarrier system for effective delivery of curcumin in cancer therapy

**DOI:** 10.1080/10717544.2016.1245365

**Published:** 2017-02-03

**Authors:** Preeti Kumari, Omkara Swami Muddineti, Sri Vishnu Kiran Rompicharla, Pratyusha Ghanta, Adithya Karthik B B N, Balaram Ghosh, Swati Biswas

**Affiliations:** Department of Pharmacy, Birla Institute of Technology and Science, Pilani, Hyderabad, India

**Keywords:** Polymeric micelles, cholesterol, curcumin, mPEG–PLA-Ch, cancer

## Abstract

Polymeric micelles have been widely explored preclinically as suitable delivery systems for poorly soluble chemotherapeutic drugs in cancer therapy. The present study reported the development of cholesterol (Ch)-conjugated poly(D,L-Lactide) (PLA)-based polymeric micelles (mPEG–PLA-Ch) for effective encapsulation and delivery of curcumin (CUR) at the tumor site. Cholesterol conjugation dramatically affected the particle size and improved drug loading (DL) and encapsulation efficiency (EE). mPEG–PLA-Ch-CUR showed bigger hydrodynamic diameter (104.6 ± 2.1 nm, and 169.3 ± 1.52 nm for mPEG–PLA and mPEG–PLA-Ch, respectively) due to increased size of the hydrophobic core. The newly developed polymer exhibited low critical micelles concentration (CMC) (25 μg/mL) which is close to lipid-based polymer, PEG-phosphatidyl ethanolamine (12.5 μg/mL) compared to mPEG–PLA (50 μg/mL). mPEG–PLA-Ch micelles exhibited relatively higher EE (93.74 ± 1.6%) and DL (11.86 ± 0.8%) compared to mPEG–PLA micelles (EE 91.89 ± 1.2% and DL 11.06 ± 0.8%). mPEG–PLA-Ch micelles were internalized by the cancer cells effectively and exhibited higher cytotoxicity compared to free CUR in both, murine melanoma (B16F10) and human breast cancer (MDA-MB-231) cells. mPEG–PLA-Ch exhibited satisfactory hemocompatibility indicating their potential for systemic application. Further, mPEG–PLA-Ch-CUR demonstrated higher rate of reduction of tumor volume in B16F10-xenografted tumor-bearing mice compared to free CUR. At the end of 22 days, the tumor reduced to 1.87-fold (627.72 ± 0.9 mm^3^ versus 1174.68 ± 1.64 mm^3^) compared to the treatment with free CUR. In conclusion, the experimental data *in vitro* and *in vivo* indicated that the newly developed CUR-mPEG–PLA-Ch micelles may have promising applications in solid tumors.

## Introduction

Cancer is a deadly disease faced by humanity which, according to the report published by WHO took a toll of 8.2 million cancer-related deaths in 2012. Anticipated rise of new cancer cases is from 14 million in 2012 to 22 million within the next two decades. As a result of intensive research in the area of anticancer drug discovery in past few decades, various potent molecules with high cytotoxic potential have been discovered. However, these drugs face challenge to be an effective treatment modality, as these drugs are cytotoxic toward normal cells as well. Therefore, there is an unmet need to improvise strategies to deliver anticancer drugs effectively and specifically to the tumor region.

In recent years, advancement in nanotechnology directed development of nanomedicines, in which drug is encapsulated in nanosized drug delivery system (Alexis et al., 2010). Nanocarriers possess distinct advantage over conventional free drug administration, as they eventually accumulate in the tumor area by a passive targeting phenomena, commonly referred to as enhanced permeability and retention (EPR) effect (Torchilin, 2011). The nanocarriers take advantage of the leaky tumor vasculature that allows nanosized carriers to escape the circulation and accumulate in the tumor micro-environment (Biswas & Torchilin, 2014). Various biocompatible self-assembled polymeric systems including liposomes, micelles; polymers of defined architectures including dendrimers; and inorganic nanoparticles including gold, silver, iron oxide and silica have been developed where poorly soluble chemotherapeutic drugs are either loaded or conjugated for their delivery to the tumor site (Kumari et al., 2016a). Nanocarriers promote solubilization, impart stability to the poorly aqueous soluble drugs and thereby improve their biopharmaceutical properties. Even though, the benefit of using nanoparticles as drug delivery system for cancer therapy is obvious, the nanocarriers have to be biocompatible, biodegradable and less immunogenic to obtain maximum therapeutic benefit.

Among many other drug delivery systems, polymeric micelles (PMs) prepared from amphiphilic block copolymers received considerable attention in drug delivery research and have been applied extensively to solubilize various poorly soluble anticancer drugs, including doxorubicin (Yokoyama et al., 1998), paclitaxel (Lee et al., 2012), cisplatin (Nishiyama et al., 2003) and methotrexate (Li & Kwon, 2000). Polyethylene glycol (PEG), a hydrophilic polymer is widely used as the outer shell for hydrophobic core in many self-assembled polymeric systems (Shuai et al., 2004; Endres et al., 2011). PEG is a biocompatible, biodegradable and nontoxic polymer with the ability to provide a stabilizing interface between the micellar core and the aqueous phase. Importantly, PEG reduces the nanoparticles (NPs) uptake by the mononuclear phagocytic system, sometime also referred to as the “stealth function” compared to the particles without PEG attachment (Gref et al., 2000). The opsonization-inhibiting property of PEG enables long circulation of NPs *in vivo* (Owens & Peppas, 2006). Polylactic acid (PLA) is a hydrophobic, biodegradable and biocompatible polymer widely used in drug delivery and bioengineering (Jain et al., 2009; Nagarwal et al., 2009). In drug delivery, the polymeric micelles constituted by the self-assembly of PEG–PLA polymer have been used as carriers for poorly water-soluble drugs (Zhang et al., 2005; Xiao et al., 2010). Genexol®-PM, developed by Samyang Genex Co. (Seoul, Korea), composed of PEG–PLA is the only clinically approved PLA-based nanomicellar chemotherapeutic, which loaded poorly soluble paclitaxel. In a recent study, we demonstrated the potential of PEG–PLA micelles to load poorly water-soluble chemotherapeutic drug, curcumin and efficiently deliver it to various cancer cell lines (Kumari et al., 2016b). However, low drug loading limits their biomedical application. The slow degradation of PLA to lactic acid by the enzyme esterase is the limitation, which prevents the use of high-molecular-weight PLA to improve hydrophobicity of the micelles (Wang et al., 2013). The inclusion of PEG in copolymer systems imparted extremely beneficial surface properties within the body because of the ability to repel proteins within aqueous environments (Andrade et al., 1993). This repulsion inhibited the adsorption of proteins to the polymer surface and, therefore, prevents many polymer–cell interactions. For example, nanoparticles made from diblock PLA–PEG copolymer have increased blood circulation times (decreased clearance) *in vivo* compared to particles made from PLA alone (Gref et al., 1994).

Among various hydrophobic moieties, cholesterol has gained considerable interest in recent years to improve hydrophobicity of drug delivery systems (Wang et al., 2010; Ha et al., 2011; Chen et al., 2013; Ma et al., 2013; Cheng et al., 2014; Yao et al., 2014). Cholesterol is an indispensible structural building block for cell membranes, responsible for membrane fluidity and permeability, intracellular transport, signal transduction and cell trafficking (Yeagle, 1985; Yeagle, 1991; Maxfield & Tabas, 2005). Due to the hydrophobicity and excellent biocompatibility, cholesterol has been extensively used to improve the hydrophobicity, biocompatibility and biodegradability of the drug delivery system without using slow-degrading polymers of high molecular weights (Zhou et al., 2009). In a study, a novel Ch-conjugated micelles, mPEG-block-poly(Ch-L-glutamate) was prepared that provided enlarged core space for loading poorly water-soluble paclitaxel (Ma et al., 2013). It has been demonstrated that chitosan conjugated to cholesterol has higher colloidal stability compared to chitosan conjugated to long alkyl chains (Ha et al., 2011). Cholesterol has been grafted to polysaccharide dextran to load anticancer doxorubicin (Yao et al., 2014).

Curcumin is a naturally occurring small molecule that demonstrated powerful anticancer activity in various preclinical studies (Naksuriya et al., 2014). CUR, bis(4-hydroxy-3-methoxyphenyl)-1,6-diene-3,5-dione is a polyphenol compound derived from the rhizome of plant *Curcuma longa.* CUR has been reported to having a wide range of pharmacological activities, such as antimicrobial, antioxidant, anti-inflammatory, antiparasitic, antimutagenic, antiHIV, and anticancer with low or no intrinsic toxicity (Kawamori et al., 1999; Anand et al., 2008; Srivastava et al., 2011). CUR could inhibit the generation, vegetation and metastasis of many tumors, including breast, pancreatic, colon, stomach, liver, cervical and epithelial cell carcinoma (Kim et al., 2011). Treatment of breast and pancreatic cancers by CUR is in phase I and II of clinical trials, respectively (Dhillon et al., 2008; Mock et al., 2015). Despite its pharmacological potentials, the application of CUR in clinic is limited due to its low aqueous solubility (0.6 μg/mL) and rapid degradation in physiological conditions (Wu et al., 2011). Wang et al. reported cleavage of the heptadienedione chain of curcumin resulting in vanillin, ferulic acid and feruloylmethane as minor products (Wang et al., 1997). However, it was found that the major product detected by Wang et al. was likely the bicyclopentadione product of autoxidative transformation of curcumin (Griesser et al., 2011). After oral administration, curcumin is metabolized by reduction and conjugation. Consecutive reduction of the double bonds in the heptadienedione chain results in the formation of di-, tetra-, hexa- and octahydro-curcumin. Reduction can occur in the gut by the NADPH-dependent reductase CurA that has been identified in intestinal Escherichia coli (Hassaninasab et al., 2011; Tan et al., 2014). After systemic absorption, alcohol dehydrogenase reduces curcumin to tetra- and hexahydrocurcumin in the liver, whereas formation of di- and octahydrocurcumin required an unidentified microsomal enzyme (Ireson et al., 2001, 2002) (Hoehle et al., 2006). The reduced metabolites, especially tetra- and hexahydrocurcumin, represent the largest portion of curcumin metabolites (Pan et al., 1999). With few exceptions, their biological activities are strongly reduced compared with those of curcumin (Aggarwal et al., 2014; Wu et al., 2014). Therefore, it is necessary to improve the solubility, stability and bioavailability of CUR to utilize it as a therapeutic candidate.

In our study, we synthesized Ch-modified mPEG–PLA polymer, where cholesterol is conjugated to the free hydroxyl group of PLA (MW ∼5970 Da). The polymer self-assembled efficiently into stable micelles with low critical micelles concentrations (CMC) and loaded hydrophobic CUR efficiently. The physicochemical characteristics of CUR-loaded mPEG–PLA-Ch micelles, including morphology, particle size, zeta potential and loading efficiency were investigated. The association of the micelles with cancer cells and cytotoxic response following CUR-mPEG–PLA-Ch treatment were analyzed in murine melanoma cells, B16F10 and human breast cancer, MDA-MB-231 cell lines. Finally, the tumor-reducing efficacy of CUR-mPEG–PLA-Ch micelles *in vivo* was evaluated.

## Materials and methods

### Materials

Methoxy poly(ethylene glycol) 5000 (mPEG), D,L-lactide, cholesteryl chloroformate, curcumin, tetrahydrofuran (THF), DAPI 4′,6-diamidino-2-phenylindole dihydrochloride (4,6- diamidino-2-phenylindole), and *para*-formaldehyde were purchased from Sigma-Aldrich Chemicals (Germany). Thiazoyl blue tetrazolium bromide (MTT), fluoromount-G and trypan blue solution were obtained from Himedia (Mumbai, India). Dialysis membrane was purchased from Spectrum Laboratories, Inc., Piscataway Township, NJ.

For cell culture, Dulbecco’s modified eagle’s medium (DMEM), growth medium RPMI-1640, penicillin–streptomycin, trypsin-EDTA and fetal bovine serum (FBS) were purchased from Himedia (Mumbai, India). All reagents were used as received and the solvents were purified according to the general procedures.

### Cell culture and animals

Murine melanoma cells, B16F10 and human breast cancer cells, MDA-MB-231 were purchased from National Center for Cell Sciences (Pune, India). Cells were grown in DMEM and RPMI-1640 medium supplemented with 10% FBS and 1% penicillin-streptomycin solution. Cells were maintained in a humidified atmosphere at 37 °C and 5% CO_2_.

Pathogen free female C57BL/6 mice of age 6–8 weeks were procured from National Center for Laboratory Animal Sciences (NCLAS), National Institute of Nutrition (Hyderabad, India). All animal studies were carried out under the guidelines compiled by the Institutional Animal Ethics Committee of the BITS Pilani University. The animals were maintained in a room (23 ± 2 °C and 60 ± 10% humidity) under a 12-h light/dark cycle. Food and water were given *ad libitum*.

### Synthesis and characterization of mPEG–PLA-ch copolymer

#### Synthesis of methoxy-polyethylene glycol–poly(lactic acid) mPEG–PLA copolymer

The methoxy-polyethylene glycol–poly(lactic acid) (mPEG–PLA) diblock copolymers were synthesized by ring opening polymerization according to our previously reported procedure (Kumari et al., 2016b). In brief, mPEG (1 g) and D,L-lactide (0.4 g) were placed in a dried polymerization tube. An appropriate amount of stannous octoate (0.008% w/w) was added as a solution in toluene. The reaction was placed in a pre-heated oil-bath at 160 °C for 6 h. After cooling to the room temperature, the resultant copolymer was dissolved in tetrahydrofuran, recovered by precipitation into an excessive solvent of ice-cold diethyl ether. The precipitant was dried and redissolved in water and kept for dialysis against water by using cellulose ester membrane (MWCO 12–14 000 Da). The product was lyophilized and stored.

#### Synthesis of cholesterol-modified methoxy-polyethylene glycol–poly(lactic acid) (mPEG–PLA-ch) copolymer

mPEG–PLA-Ch copolymers were synthesized through a coupling reaction between the hydroxyl group at the end of mPEG–PLA and cholesteryl chloroformate. In a typical run, into the solution of mPEG–PLA (500 mg, 0.17 mmol) and triethylamine (17 μL, 0.67 mmol) in 4 mL of DCM at 0 °C and under N_2_ atmosphere, cholesteryl chloroformate (100 mg, 0.67 mmol) was added drop wise at 0 °C. The mixture was stirred for 24 h at room temperature, concentrated and precipitated into diethyl ether. The precipitate was dried, suspended in water and kept for dialysis against water by using cellulose ester membrane (MWCO 12–14 000 Da). The product was lyophilized to yield white fluffy solid.

#### Fourier transform infrared spectroscopy (FTIR)

FTIR spectra were recorded by using KBr pellets on a FTIR (Jasco-4200, Japan) at room temperature.

#### Nuclear magnetic resonance spectroscopy (NMR)

^1^H NMR spectra was recorded on a Bruker spectrometer (AVANCE model, Germany) operating at 300 MHz at room temperature. The compound was dissolved in CDCl_3_ at concentration of 5 mg/mL.

#### Critical micelle concentration (CMC)

A steady-state pyrene fluorescence method was used to determine the CMC of the copolymers (Song et al., 2011). Fluorescence spectra were recorded on a spectrophotometer (Spectramax™ M4, Multi detection Reader). Pyrene was used as a hydrophobic fluorescence probe. Aliquots of pyrene solutions (50 μL; 10 mg/mL dissolved in chloroform) were added to the micellar solutions used at the concentrations range of 3.125–150 μg/mL. The mixtures were stirred overnight for solubilization and incorporation of pyrene in the micelles. The following day, the solutions were filtered before spectral analysis. The emission wavelength was 390 nm and the excitation spectra were recorded ranging from 300 nm to 350 nm with both bandwidths set at 5 nm. A CMC value was determined from the ratios of pyrene intensities at 337 (I337) and 334 (I334) nm and calculated from the intersection of two tangent plots of I337/I334 versus log concentrations of copolymers.

### Preparation and characterization of CUR-loaded polymeric micelles

CUR-loaded micelles were prepared by a thin film hydration method (Wei et al., 2009). Briefly, the copolymer, mPEG–PLA-Ch (10 mg) was dissolved in 1 mL of chloroform. A certain amount of CUR from the CUR solution (2.5 mg in 0.1% acetic acid methanol solution) was then added. The mixture was rotary evaporated resulting in the formation of yellowish thin layer of uniform film on the wall of the flask. The thin film was hydrated using 1 mL PBS, pH 7.4. The solution was centrifuged (13 500 g, 4 °C) to remove the unincorporated drug. Bank micelles were prepared following similar approach.

The drug-loaded micelles solution was added to 80% (v/v) ethanol to disrupt the micelles core-shell structure and to dissolve CUR released from the micelles. Through stepwise dilution, a solution of CUR with UV absorbance at a range of 0.2–0.8 at 420 nm was prepared. The CUR content in the drug-loaded micelles was determined using the UV–vis spectrophotometer (Spectramax™, microplate reader, Molecular Devices, Winooski, VT) at 420 nm. The drug-loading content (DL) and drug encapsulation efficiency (EE) were calculated based on the following formula,

$$\mathrm{EE}\%\;=\;\frac{\mathrm{Weight}\;\mathrm{of}\;\mathrm{the}\;\mathrm{drug}\;\mathrm{in}\;\mathrm{micelles}}{\mathrm{Weight}\;\mathrm{of}\;\mathrm{the}\;\mathrm{feeding}\;\mathrm{polymer}\;\mathrm{and}\;\mathrm{drug}}\times 100$$

$$\mathrm{DL}\%\;=\;\frac{\mathrm{Weight}\;\mathrm{of}\;\mathrm{the}\;\mathrm{drug}\;\mathrm{in}\;\mathrm{micelles}}{\mathrm{Weight}\;\mathrm{of}\;\mathrm{the}\;\mathrm{feeding}\;\mathrm{drug}}\times 100\;$$

#### Particle size and zeta potential analyses

Particle size, zeta potential and polydispersity index (PDI) of blank and CUR-loaded mPEG–PLA-Ch micelles were determined by dynamic light scattering (DLS, Zetasizer™ ZEN 3600 instrument, Malvern Instruments Ltd., UK). The micelles were suspended in deionized water before measurement under a fixed scattering angle of 90° at 25 °C. Results were expressed as mean ± standard deviation (SD). All the measurements were analyzed in triplicates.

#### Transmission electron microscopy (TEM) studies

Transmission electron microscope (TEM, JEM-1200EX, JEOL, Tokyo, Japan) was used for morphological observation. One drop of micelles solution was placed on a carbon film-coated copper grid. After negative staining with 2% w/v uranyl acetate for 20 s, the excess solution was absorbed by filter paper and dried in air before analysis.

#### Differential scanning calorimetry (DSC)

mPEG–PLA-Ch, free curcumin and curcumin encapsulated in mPEG–PLA-Ch micelles were analyzed using DSC (DSC 60, Shimadzu, Japan). About 1 mg of each sample was put in the aluminum pan and the lid of the pan was penetrated to form a small hole. Samples were heated from room temperature to 250 °C at the rate of 10 °C per minute under nitrogen atmosphere at a flow rate of 20 mL per minute.

### Central composite factorial design (CCD)

After opting for the most important factors influencing the physicochemical properties of the produced curcumin-loaded micelles, a two-factor, five-level CCD was developed to explore the optimum levels of these variables. This methodology consisted of two groups of design points, including two-level factorial design points, axial or star points and center points. Therefore, two selected independent variables (amount of copolymer (X1) and amount of drug (X2)) were studied at five different levels coded as −α, −1, 0, 1 and  + α. The value for alpha (1.414) was intended to fulfill the rotatability in the design. Physicochemical properties of the produced micelles, that is, DL (Y1), EE (Y2) and Z_avg_ (Y3) were selected as dependent variables. The coded and actual values of the variables are given in Table 1. According to the CCD matrix generated by Design-Expert software (Trial Version 10, Stat-Ease Inc., MN), a total of nine experiments were constructed.

**Table 1.** Independent variables and their levels of experiment design.

	Levels				
Independent variables	−1.414	−1	0	1	1.414
X1: Amount of copolymer	2.92893	5	10	15	17.0711
X2: Amount of drug	0.37868	1	2.5	4	4.62132
Dependent variables	Desired				
*Y*1: Drug loading (DL%)	Maximize				
*Y*2: Encapsulation efficiency (EE%)	Maximize				
*Y*3: Particle size (nm)	Minimize				

Optimization was performed by using a desirability function to obtain the optimal points. Concerning the predetermined constraints, DL and EE was in maximum level, and particle size was in their minimum levels (Varshosaz et al., 2010). The picked optimal formulation was prepared for further evaluation of the physicochemical characteristics of micelles.

### Hemocompatibility and release profile of the mPEG–PLA-ch micelles

The hemolysis assay procedure was modified from previously described method (Meng et al., 2011). Heparinized rat erythrocytes were separated from 5 mL of rat blood by centrifugation at 3000 rpm for 30 min and washed with physiological saline to achromatic color for supernatant solution. After centrifugation, cells were mixed with normal saline to prepare 5% RBC solution. Further, 100 μL of purified 5% RBC solution was incubated with 900 μL of mPEG–PLA-Ch in PBS at various concentrations for 1 h at 37 °C. The copolymer concentration range was set from 0.5–10 mg/mL. Physiological saline and Triton-X 100 (1% solution) were used as negative and positive control, respectively. Samples were centrifuged and hemolysis was quantified by measuring released hemoglobin (Hb). The absorbance of Hb in the supernatant at 576 nm was measured by UV–vis spectrophotometer (Spectramax™, microplate reader, Molecular Devices).

The degree of hemolysis was determined on the basis of absorbance at 576 nm and calculated from the following formula (Dutta & Dey, 2011):

$$\mathrm{Hemolysis}\;\left(\%\right)=\frac{{\mathrm{Abs}}_{\mathrm{sample}}{\;-\;\mathrm{Abs}}_{\mathrm{negative}\;\mathrm{control}}}{{\mathrm{Abs}}_{\mathrm{positive}\;\mathrm{control}}-{\mathrm{Abs}}_{\mathrm{negative}\;\mathrm{control}}}\times 100$$

where Abs_sample_, Abs_negative_ and Abs_positive_ were the absorbance of copolymer sample, physiological saline (0% hemolysis) and Triton-X 100 (100% hemolysis). Data were taken from three independent experiments.

*In vitro* drug release profile of CUR from CUR-mPEG–PLA-Ch micelles was done by dynamic dialysis method (Wu et al., 2013). One milliliter of CUR solution (100 μg/mL in propylene glycol) and 1 mL of CUR-mPEG–PLA-Ch micelles solution were put into dialysis bags (Spectrum Laboratories, Inc., Piscataway Township, NJ) of MWCO 2000 Da. The bags were placed into 40 mL of phosphate-buffered solution (PBS) (pH 7.4) containing 0.1% (w/v) Tween 80 to maintain a constant sink condition. This was kept shaking on an orbital shaker at 100 rpm at 37 ± 0.5 °C. At definite time intervals, 1 mL of the released medium was withdrawn. The same volume of fresh release mediums was supplemented to maintain a constant volume. The release amount of drug was quantified spectrophotometerically at a wavelength of 420 nm. All experiments were performed in triplicates. The release rate was calculated, and the results were expressed as mean ± SD.

### *In vitro* cellular uptake

The cellular uptake of CUR-loaded micelles was evaluated by using flow cytometry and fluorescence microscopy. For flow cytometry experiments, B16F10 and MDA-MB-231 cells were seeded on 6-well plates at 4 × 10^5^ cells/well and incubated for 24 h. Prior to adding the micelles solution, the culture medium (1 mL) was replaced with fresh medium. Then, the cells were treated with free CUR and CUR-mPEG–PLA-Ch micelles (CUR 50 and 100 μg/mL) for 1 and 4 h, respectively. After incubation, the cells were washed twice with PBS, detached with trypsin-EDTA and resuspended in PBS, pH 7.4 (200 μL) for flow cytometer analysis (Amnis Flowsight, United States). The data presented are the mean fluorescent signals for 10 000 cells.

For fluorescence microscopy experiments, the cells were first seeded on microscope slides and incubated with free CUR and CUR-loaded micelles in the same way as that in the flow cytometry experiments. After incubation, the culture medium was removed and the cells on microscope slides were washed with PBS, fixed with 4% paraformaldehyde, stained with DAPI and mounted cell side down on superfrost microscope slides with fluorescence-free glycerol-based mounting medium (Fluoromount-G; Sigma Aldrich). Fluorescence images of cells were obtained with an Inverted Fluorescence microscope (Leica Microsystems, Germany). The images of the cells were taken using FITC filter (λex 495 and λem 520 nm). The LSM picture files were analyzed by using Image J software, NIH, Rockville, MD.

### *In vitro* cytotoxicity

Cytotoxicity of CUR-mPEG–PLA-Ch micelles was investigated by the MTT assay. About 100 μL of B16F10 and MDA-MB-231 cell lines were seeded in 96-well plates at the density of 1 × 10^4^ cells/well and 5 × 10^3^ cells/well with DMEM and RPMI-1640, respectively, and incubated overnight to allow cell attachment. The old media were discarded and the cells were incubated with CUR-mPEG–PLA-Ch micelles, blank micelles and free curcumin for 6 h in serum-free media and 24 h in complete growth media, respectively. After 6 h, the medium was removed and the wells were incubated for additional 24 h with fresh medium. The following day, 50 μL of MTT solution (5 mg/mL) prepared in the serum/phenol red-free DMEM and RPMI-1640 was added to each well. The plates were further incubated for 4 h. Finally, MTT in medium was removed and 150 μL of DMSO was then added to each well to dissolve the formazan crystals. Each sample was tested in six replicates per plate and assayed at 570 nm wavelength using a microplate reader (Spectramax™, microplate reader, Molecular Devices, Winooski, VT) with a reference wavelength of 630 nm. Cell viability was calculated by the followed equation:

Cellviability(%)=ABS of sample/ABS of control ×100

where ABS of sample is the absorbance of the transformed MTT in cells incubated with the formulations while the ABS of control is the absorbance of transformed MTT in cells incubated with the culture medium only (positive control).

### Antitumor activity assay

C57BL/6 mice were inoculated subcutaneously with murine melanoma cells, B16F10 (5 × 10^5^ cells in 100 μL of PBS). Tumors were allowed to grow for 2–3 weeks to reach proliferative phase (approximately 50–100 mm^3)^. Subsequently, CUR-loaded mPEG–PLA-Ch micelles in PBS (pH 7.4) were injected intraperitoneally once at 2-day interval (CUR dose. 25 mg/kg). The micelles were diluted to a concentration of 10 mg of total copolymer or 500 μg of CUR/mL of micelle solution so that the mice (approximately 20 g) received ∼200 μL of micelle preparation intraperitoneally. Groups of mice were as follows: (i) saline (the control group); (ii) free curcumin and (iii) CUR-mPEG–PLA-Ch (*n* = 4 in each group). Each sample was intraperitoneally injected every two days for 22 days (5 times). Tumor size and animal body weight were measured every two days during the study. The tumor volume and body weight were recorded for all tumor-bearing mice for 22 days until the tumor size of animals in the control and CUR-mPEG–PLA-Ch groups reached 1500 mm^3^, after which animals were sacrificed in CO_2_ chamber. The length and width of the tumors were measured by Vernier caliper and the tumor volume was calculated using the following formula:

$$V={a\times b}^{2}/2$$

where *a* and *b* denote the long and short diameters of the tumor, respectively.

The postmortem tumor weight was taken after washing the tumors with PBS. The tumors were embedded in tissue-freezing media and stored at  −80 °C. For tumor histology, tumor slices (5 μM) were cryosectioned and stained with the Terminal Deoxynucleotidyl Transferase Biotin-dUTP Nick End Labeling (TUNEL) assay following the manufacturer's protocol and examined under a fluorescence microscope equipped with green filter.

### Statistical analysis

The data were assessed for statistical significance using Student’s paired *t*-test. All numerical *in vitro* data are expressed as mean ± SD, *n* = 6. *In vivo* data are expressed as mean ± SEM, *n* = 4 in each group. Two-way analysis of variance (ANOVA) followed by the Bonferroni's *post hoc* test were conducted for all paired groups using Graph Pad prism 5 software (GraphPad Software, Inc.; San Diego, CA). Any *p* value less than 0.05 was considered statistically significant.

## Result and discussion

### Synthesis and characterization of mPEG–PLA-ch copolymers

**Scheme 1.**
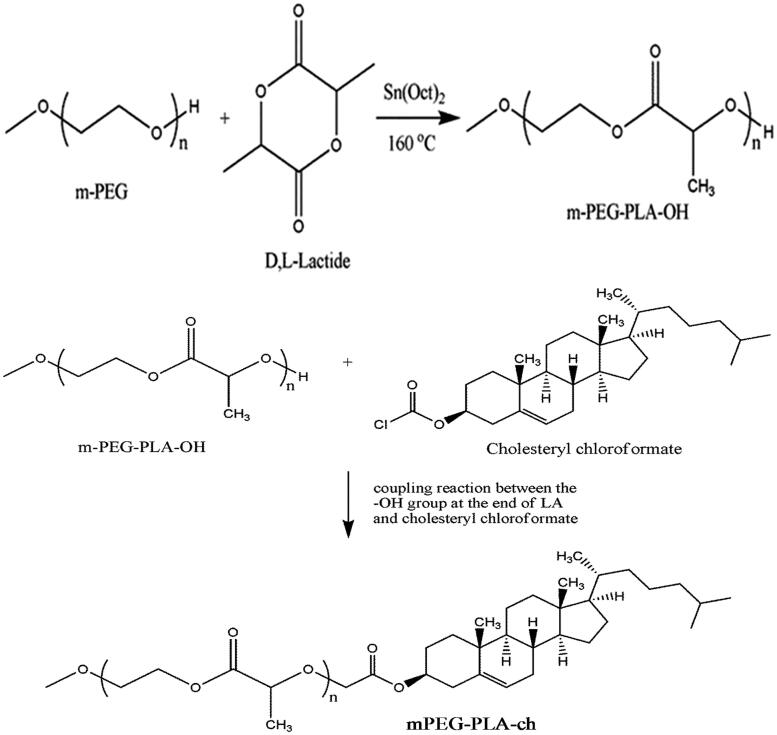
Detailed synthetic route of mPEG–PLA-Ch copolymers.

mPEG–PLA-Ch block copolymers were prepared through two reaction steps. In the first step mPEG–PLA diblock copolymer was prepared by ring opening polymerization reaction of mPEG and D,L-lactide by using stannous octoate as the catalyst followed by modifying the terminal hydroxy group of mPEG–PLA with cholesteryl chloroformate (Scheme 1). The final cholesterol-conjugated mPEG–PLA polymer was purified by precipitation and subsequent membrane dialysis to completely remove the unreacted cholesteryl chloroformate. The final polymer was obtained as a fine white powder as 71% yield.

**Figure 1.**
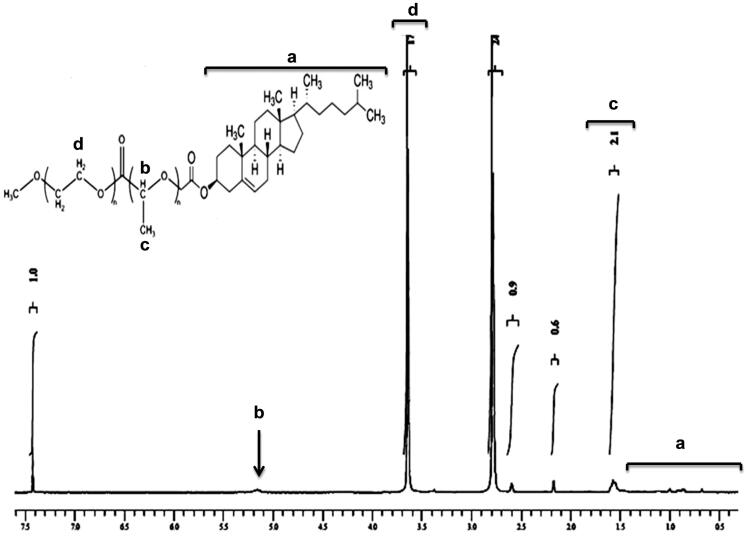
^1^H NMR spectrum of mPEG–PLA-Ch copolymers.

The ^1^H HMR spectrum of mPEG–PLA-Ch copolymer is shown in Figure 1. The peaks at 1.6 and 5.1 ppm were assigned to CH_3_-O- and multiplet of –CH of PLA, respectively. Moreover, the peak at 3.6 ppm from the methylene protons of PEG (-OCH_2_-CH_2_-) can also be observed. After conjugation with cholesteryl chloroformate, ^1^H NMR spectrum of the polymer exhibited two singlets at 0.68 and 1.02 ppm, and two doublets at 0.85 and 0.91 ppm, which are assigned to four kinds of methyl groups of the cholesteryl moieties. The graph and the detail analysis of the IR of mPEG–PLA-Ch were reported in the supplementary section.

### Preparation and characterization of CUR-loaded polymeric micelles

For the efficient encapsulation of curcumin (CUR), mPEG–PLA-Ch copolymer was used based on its solid state solubility and compatibility. We adopted the thin film hydration method to prepare curcumin loaded mPEG–PLA-Ch micelles. Solubilization of hydrophobic anticancer drugs and development of biocompatible drug delivery systems are the prime aim of drug delivery research (Shaikh et al., 2009). Thin film hydration method is most often used due to its simplicity and practicability, and its ability to yield small and uniform particles.

**Figure 2.**
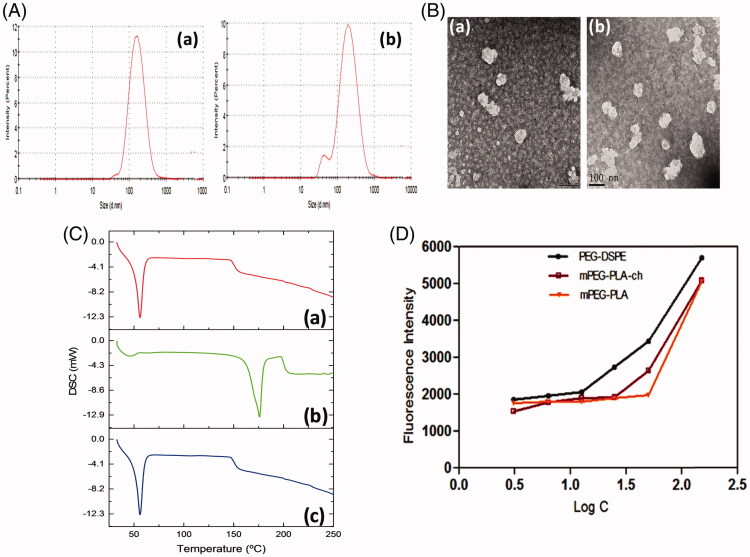
Physicochemical characterization of polymeric micelles. A. Particle size distribution of mPEG–PLA-Ch (a) and CUR-mPEG–PLA-Ch micelles (b) measured by light scattering method; B. Transmission electron micrograph of the mPEG–PLA-Ch (a) and CUR-mPEG–PLA-Ch micelles (b); C. Differential scanning calorimetry thermograms of the CUR-mPEG–PLA-Ch (a), free CUR (b), and mPEG–PLA-Ch (c); D. Determination of critical micelles concentrations of mPEG–PLA-Ch compared to PEG-DSPE.

For physicochemical characterization of polymeric micelles, particle size, size distribution and surface charge were measured (Figure 2). The mean particle diameters of blank and CUR-loaded mPEG–PLA-Ch micelles measured from the dynamic light scattering were 169.3 ± 1.52 and 189.9 ± 0.36 nm with polydispersity index of 0.300 and 0.488, respectively, as shown in Figure 2(A). The drug was loaded into micelles, resulting in an increase in the mean particle diameter. Zeta potentials of blank and CUR-loaded mPEG–PLA-Ch micelles were −12.3 ± 0.59 mV and −18.2 ± 0.72 mV, respectively. Surface morphology of the CUR-loaded mPEG–PLA-Ch micelles was visualized by TEM (Figure 2B). Characterization by DSC gives an insight into the melting and recrystallization behavior of the crystalline materials loaded in the micelles. Figure 2(C) showed DSC curves of curcumin, mPEG–PLA-Ch copolymer and CUR-loaded mPEG–PLA-Ch micelles. The pure curcumin displayed a single sharp endothermic peak at 171.93 °C. However, no such peak depression was observed in CUR-loaded micelles, suggesting a high distribution of CUR throughout the polymer matrix in micelles. This data also indicated that the CUR was present in amorphous stage in the mPEG–PLA-Ch micelles.

The ability of the polymer to form stable polymeric micelles was assessed by determining CMC. Pyrene method was used to measure the CMC of the micelles (Song et al., 2011). With the logarithm of concentration of micelles as the abscissa and the fluorescence intensity as the ordinate, the CMC of the mPEG–PLA-Ch micelles was 25 μg/mL gained from intersection of the two tangents to scatter diagram (Figure 2D). The CMC of novel micellar system was comparable to standard micelle-forming polymer, PEG-DSPE (CMC for PEG-PE 12.5 μg/mL in the assay system). Modification with cholesterol improved the hydrophobicity of the core in mPEG–PLA micelles resulting in superior micellization ability as judged by their low CMC value compared to mPEG–PLA micelles (reported CMC of mPEG–PLA 50 μg/mL based on our previous data) (Kumari et al., 2016b). Because of the low CMC, the micelles had high stability and ability to maintain integrity even after extreme dilution in the systemic circulation.

The samples were analyzed spectrophotometerically to determine CUR fluorescence at 420 nm. The blank sample had no absorbance at this wavelength in the CUR-concentration range of 2–20 μg/mL, the following standard regression equation was obtained, A = 0.1473B + 0.0086, R2 = 0.9998 (*n* = 3). The average EE and DL of the optimized CUR-mPEG–PLA-Ch formulation were 93.74 ± 1.62% and 11.86 ± 0.84%, respectively compared to mPEG–PLA micelles (EE 91.89 ± 1.2% and DL 11.06 ± 0.8%) (Kumari et al., 2016b).

### Statistical analysis of experimental data by design-expert software

The results of the experimental design indicated that this system was highly influenced by the amount of copolymer and drug ratio which resulted in high drug EE and small particle sizes for the preparation of micelles. Hence, the two factors were conducted as variables for optimization through CCD at five experimental levels. Amount of copolymer was as X1 ranging from 2.9 to 17.07 mg, and the amount of CUR was as X2 in the range from 0.3 to 4 mg. The experimental design and results were shown in Table 2. The quadratic polynomial equation was obtained from the software is as follows:

(1) $$Y\left(\mathrm{Zavg}\right)=152.578-16.757X_{1}+17.294X_{2}+1.120X_{1}^{2}\;+8.367X_{2}^{2}-4.2373X_{1}X_{2}$$

(2) $$Y\left(\mathrm{DL}\%\right)=17.428-0.92323X_{1}+0.13351X_{2}\;+0.021972X_{1}^{2}-0.41653X_{2}^{2}+0.13083X_{1}X_{2}$$

(3) $$Y\left(\mathrm{EE}\%\right)=28.124+8.7588X_{1}+46.609X_{2}-0.36193X_{1}^{2}\;-11.088X_{2}^{2}+0.4591X_{1}X_{2}$$

**Table 2.** Central composite design with code values and observed values.

	Code value of variables		Actual value of variables		Observed values		
No.	*X*1	*X*2	Amount of copolymer (mg)	Amount of curcumin (mg)	Zavg	EE %	DL %
1	0	1.414	10	4.62132	168.3	28.1799	8.905
2	−1	1	5	4	212.4	28.1708	8.07593
3	−1	−1	5	1	86.71	54.4213	10.7369
4	1.414	0	17.0711	2.5	136.2	81.9705	11.901
5	1	1	15	4	82.78	47.5852	10.0179
6	0	−1.414	10	0.37868	114	36.7897	13.3545
7	1	−1	15	1	84.21	60.0626	8.75391
8	0	0	10	2.5	185.4	93.1609	11.3941
9	−1.414	0	2.92893	2.5	182.8	35.4133	16.3045

where *X*1 represents the amount of polymer, *X*2 represents the amount of curcumin.

First, the polynomial equation depicting the dependent and independent variables were analyzed. Then, further optimization and validation process by means of the design expert software was undertaken with desirable characteristics to probe the optimal formula solution of micelles which depended on the prescriptive criteria of maximum DL and EE, minimum particle size. The composition of optimum formulation was determined as 10 mg of copolymer and 2.5 mg of CUR, which fulfilled the requirements of optimization. At these levels, the predicted values of *Y*1 (DL), *Y*2 (EE) and *Y*3 (*Z*_avg_) were 93.16%, 11.39% and 185.4 nm, respectively. Therefore, in order to confirm the predicted model, a new batch of micelles according to the optimal formulation factors levels was prepared. The observed optimized formulation had DL of (11.86 ± 0.84%), EE of (93.74 ± 1.62%) and *Z*_avg_ of (189.9 ± 0. 36 nm), which were in good agreement with the predicted values shown in Table 3. A comparison between these observed results and theoretical predictions indicated the reliability of CCD used in predicting the desirable micellar formulation.

**Table 3.** Observed results and predicted results of Zavg, DL and EE based on the optimal preparation technology of CUR-mPEG–PLA-Ch.

	Zavg (nm)	DL%	EE%
Theoretical	185.4	11.39	93.16
Practical	189.9 ± 0. 36	11.86 ± 0.84	93.74 ± 1.62

**Figure 3.**
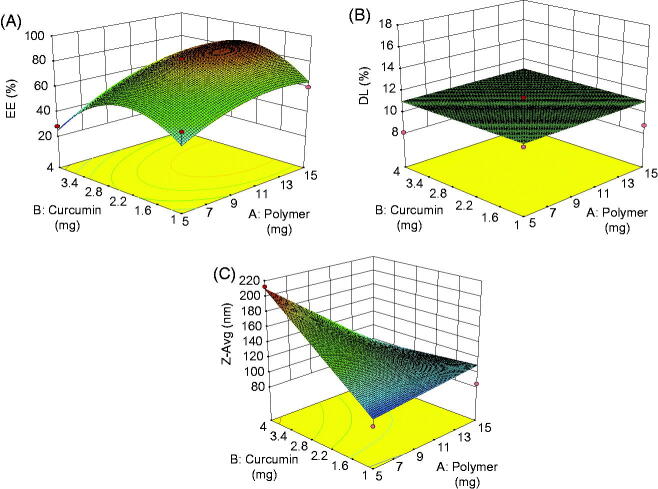
The response surface plots of DL (A) EE (B), and *Z*_avg_ (C) obtained from central composite design.

Response surface analyzes were also plotted in three-dimensional model graphs for the optimization of nanocarriers with suitable and satisfied physicochemical properties. The three-dimensional response surface plots for DL, EE and particle size were presented in Figure 3(A–C). The response surface plots were used to describe the interaction and quadratic effects of two independent variables on the responses or dependent variables.

### Hemocompatibility and release profile of the mPEG–PLA-ch micelles

**Figure 4.**
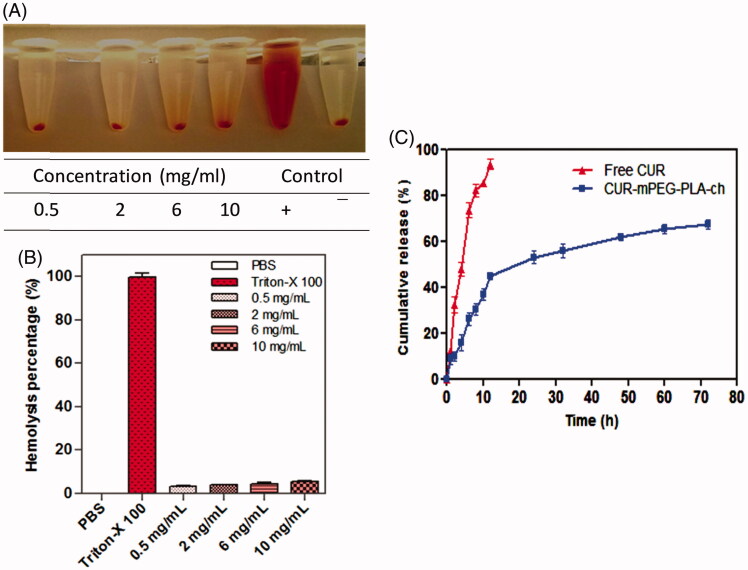
Assessment of hemocompatibility of the polymer (A and B) and the release of loaded CUR (C). A and B. Percentage of hemolysis of mPEG–PLA-Ch at 0.5, 2, 4, 6 and 10 mg/mL concentration at pH 7.4; C. *In vitro* CUR-release profile from free CUR and CUR-mPEG–PLA-Ch micelles in media (PBS, pH 7.4) (data are presented as mean ± SD (*n* = 3)).

To explore the compatibility of the copolymers with blood components, hemolytic assays were performed for the polymer, mPEG–PLA-Ch in PBS pH 7.4. Freshly isolated rat red blood cell (RBC) suspension (5% v/v) was added to PBS, 1% Triton X-100 and polymers with a final concentration of 0.5, 2, 6 and 10 mg/mL and incubated for 60 min at 37 °C. The pictures associated with the hemolytic experiments are presented in inset of Figure 4(A). As seen, clearly none of the samples exhibited any hemolysis. To quantify the hemolytic activity of polymer samples for each treatment, the percentage of cell lysis relative to the untreated cell (% control) was determined by measuring the absorbance (576 nm) of the supernatant. Hemolytic activity (in %) of the copolymer along with the  positive and  negative control is presented in Figure 4(B). Based on the previous studies, any sample with less than 5% hemolysis ratio is regarded as nontoxic (Rao & Sharma, 1997). In the present investigation, it is observed that the copolymer mPEG–PLA-Ch at a concentration of 0.5, 2, 6 and 10 mg/mL exhibited 3.2 ± 1.07, 3.59 ± 0.96, 3.98 ± 0.64 and 4.74 ± 0.21% hemolysis, respectively, whereas mPEG–PLA copolymer showed 4.86 ± 0.92% hemolysis at highest concentration of 10 mg/mL. Therefore, with cholesterol modification in side chain of copolymers did not exhibit any significant lysis to RBC membrane and hemolytic activity of the copolymers was independent on the hydrophobic composition or hydrophobic chain length to mPEG–PLA-Ch.

The release of curcumin from the mPEG–PLA-Ch micelles was investigated in PBS at 37 °C. The drug release was determined at pre-determined intervals of up to 72 h. The cumulative percentage release of CUR was shown in Figure 4(C). Initial release of curcumin from the formulations is considered to be a burst release of deposited or weakly bound drug on the surface of the micelles (Mittal et al., 2007). The drug release percentage from the CUR propylene glycol solution and CUR-mPEG–PLA-Ch micelles was 80.16% and 47.42% of the encapsulated drug during the first 12 h, respectively. The release percentage of CUR reached 91.69% and 69.83% within following 12 h, respectively. However, only 9.73% of CUR was released from the CUR-mPEG–PLA micelles within the first 6 h, while about 64.24% of CUR was released from the propylene glycol solution during the same time period (Kumari et al., 2016b). Therefore, most of the CUR was embedded in the hydrophobic core by hydrophobic interaction, and the amount of CUR in the surface of the nanoparticles was insignificant. The released mechanism of CUR from micelles might be related to the drug diffusion and the disintegration of polymer material (Liu et al., 2001; Ruan & Feng, 2003). The drug release process was mainly as follows, firstly, the media gradually got into the micellar interior to dissolve CUR and the dissolved drug spread to the media slowly. Then, the carrier material was corroded and degraded, and the CUR was released with a slow rate.

### Cellular uptake of CUR micelles

**Figure 5.**
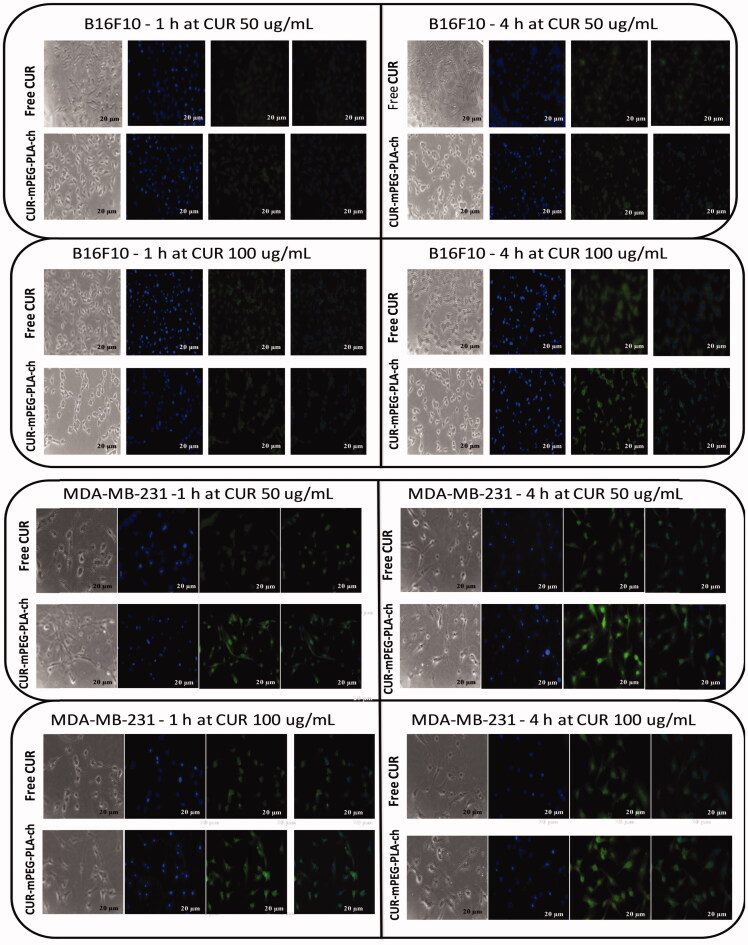
Fluorescence microscopy images of cancer cells (B16F10, and MDA-MB-231) to assess cellular uptake of CUR-loaded polymeric micelles. A. B16F10 cells treated with free CUR and CUR-mPEG–PLA-Ch at CUR concentration of 50 and 100 μg/mL; B. fluorescence micrograph of MDA-MB-231 cells following the same treatment as B16F10 cells. Blue and green signals present cell stained by DAPI and CUR fluorescence in cells, respectively (Scale bar = 20 μm).

Free CUR and CUR-loaded micelles-treated cancer cells were visualized under fluorescence microscope to assess cellular uptake of the nanocarriers (Figure 5). The result demonstrated that these micelles were actively taken up by B16F10 (Figure 5A) and MDA-MB-231cells (Figure 5B) as seen by green fluorescence within the cells. No fluorescence was observed in cells treated with blank micelles. CUR-loaded micelles could rapidly accumulate in the cytosol of cells in 1 h revealed by bright green fluorescence compared to the treatment with free CUR which resulted in less intense fluorescence in the cytosol. Further, intensity of green fluorescence in cytosol after micelles treatment was much brighter after 4 h compared to 1 h treatment.

**Figure 6.**
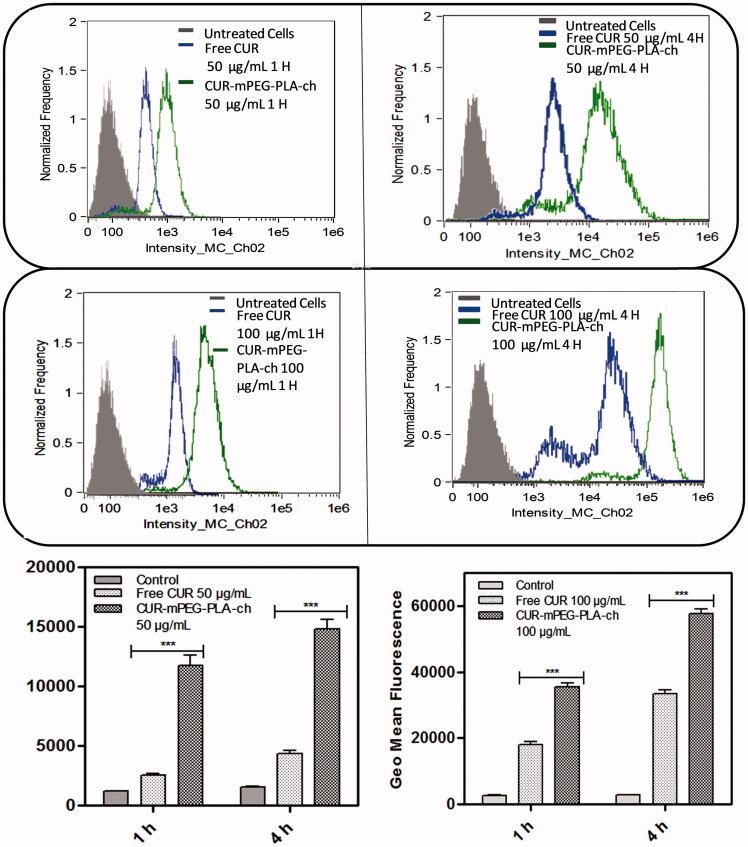
Quantification of cellular association of Free CUR and CUR-mPEG–PLA-Ch micelles by flow cytometry in B16F10 cells. The cell-associated CUR fluorescence was measured. The figure represents the histogram plots and comparison of the geometric mean of fluorescence of the cells following 1- and 4-h treatment with Free CUR or CUR-mPEG–PLA-Ch. The data are mean ± SD, averaged from three separate experiments. The significance of difference between the mean was analyzed by Student’s *t*-test, ***p  < 0.001.

**Figure 7.**
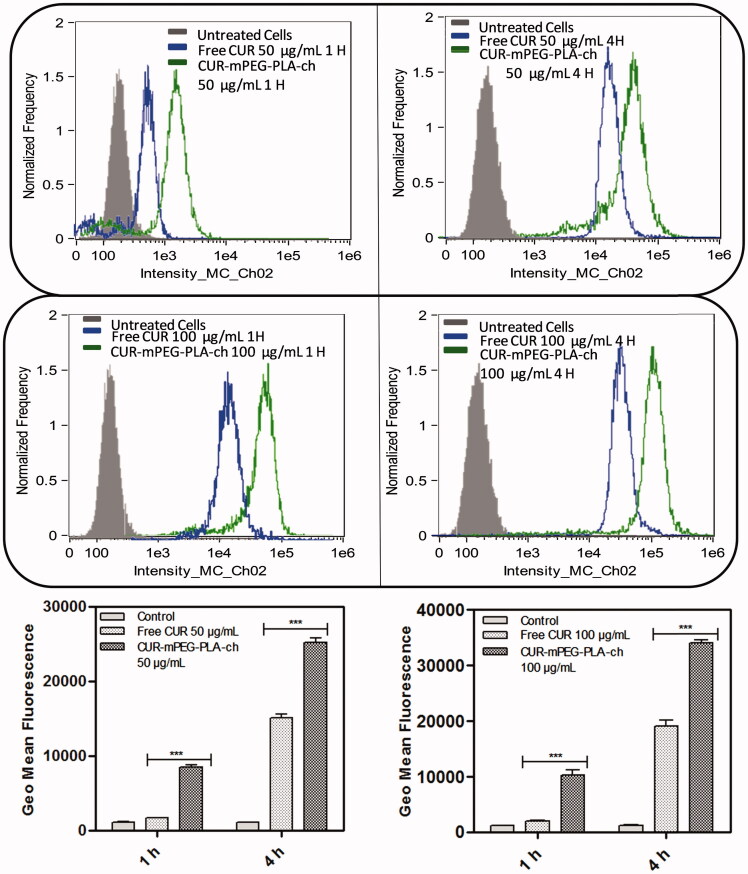
Quantification of cellular association of Free CUR and CUR-mPEG–PLA-Ch micelles by flow cytometry in MDA-MB-231 cells. The cell-associated CUR fluorescence was measured. The figure represents the histogram plots and comparison of the geometric mean of fluorescence of the cells following 1- and 4-h treatment with Free CUR or CUR-mPEG–PLA-Ch. The data are mean ± SD, averaged from three separate experiments. The significance of difference between the mean was analyzed by Student’s *t*-test, ***p< 0.001.

The intensity of fluorescence was quantified by flow cytometry analysis. The study was performed on both the cell lines in dose- (50 and 100 μg/mL) and time (1 and 4 h)-dependent manner (Figures 6 & 7). Time- and dose-dependent cellular association of nanocarriers were observed. Intensity of fluorescence (represented as the geometric mean of fluorescence) was much stronger for the cells treated with CUR-loaded mPEG–PLA-Ch micelles compared to the free CUR treatment and CUR-mPEG–PLA micelles (Kumari et al., 2016b). Significantly lower intensity of cellular fluorescence following free CUR treatment could be due to the absence of a carrier system as well as the instability of the free CUR.

### Cytotoxicity studies

**Figure 8.**
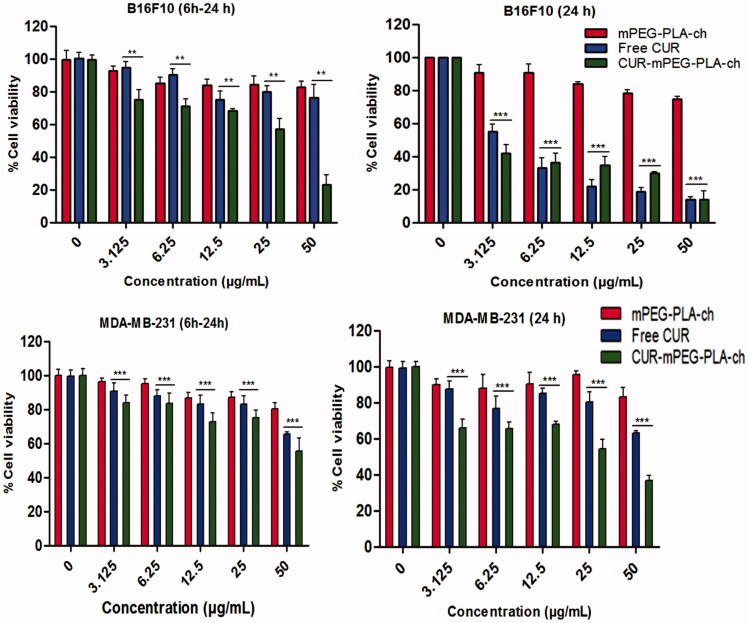
Measurement of *in vitro* cytotoxicity of blank micelles, free CUR and CUR-mPEG–PLA-Ch micelles by MTT assay against B16F10 and MDA-MB-231 cell lines. The CUR concentration range was 0–50 μg/mL, and the period of incubation was 6 and 24 h. The cells undergoing 6-h treatment were incubated for additional 24 h before performing MTT assay to determine cell viability. The significance of difference between the mean was analyzed by Student’s *t*-test, ***, ** indicates *p* < 0.001 and 0.01, respectively (data are presented as mean ± SD (*n* = 3).

The *in vitro* cytotoxicity of both the murine and human cancer cells following treatment with CUR, either free or in micellar form was investigated by MTT assay. Blank micelles exhibited no cytotoxicity as the cellular viability was above 90% at all concentrations used during experiment (Figure 8). These results indicated that the cytotoxicity following CUR-mPEG–PLA-Ch treatment was solely due to the efficient delivery of CUR. As stated earlier, two treatment protocols were followed: (i) treatment for 6 h followed by 24-h incubation before measurement of cell viability; (ii) treatment for 24 h before measurement of cell viability. The result demonstrated that the free CUR decreased the viability of B16F10 cells significantly when incubated for 24 h compared to 6-h treatment (Figure 8); however, the same treatment with free CUR showed no time-dependent cytotoxicity in MDA-MB-231 cell lines (Figure 8). The cytotoxic effect of CUR was more prominent in B16F10 cells compared to MDA-MB-231 cell line. CUR-mPEG–PLA-Ch micelles demonstrated higher cellular toxicity compared to free CUR at the highest tested CUR dose of 50 μg/mL following the incubation period of 6 h (30.11 ± 2.3% versus 73.13 ± 3.7% cell viability for CUR-mPEG–PLA-Ch versus free CUR, respectively). However, no such significance in difference in cell viability was observed in B16F10 cell lines following 24-h incubation with the same dose of 50 μg/mL. Following 6-h incubation, at CUR-concentration of 50 μg/mL, CUR-micelles produced significantly higher cellular toxicity compared to free CUR (cell viability of 55.26 ± 3.7% versus 66.84 ± 2.4%, for CUR-micelles and free CUR, respectively). Similar trend in cell viability was observed following 24-h incubation with cell viability of 35.46 ± 4.3% for CUR-mPEG–PLA-Ch compared to 62.75 ± 0.9% for free CUR at CUR concentration of 50 μg/mL.

As shown in Figure 8, CUR-mPEG–PLA-Ch treatment was more effective in killing cancer cells except at 24-h time point in B16F10 cell line. This could be due to incomplete release of the loaded drug from the formulation. Further, previous studies by other groups indicated that the IC50 value varied in different cell lines with the same treatment (Wichitnithad et al., 2011). Concerning B16F10 cell line, similar result was reported by Anuchapreeda et al. where they have shown that the encapsulation of CUR in nanoemulsion could not reduce the IC50 value compared to the administration of free CUR to B16F10 cells (Anuchapreeda et al., 2011). Variability in IC50 values following CUR administration in different leukemic and B16F10 cell lines was demonstrated by this study. Therefore, *in vitro* cytotoxicity result might not be a true prediction of the *in vivo* therapeutic activity.

### Assessment of *in vivo* therapeutic efficacy

**Figure 9.**
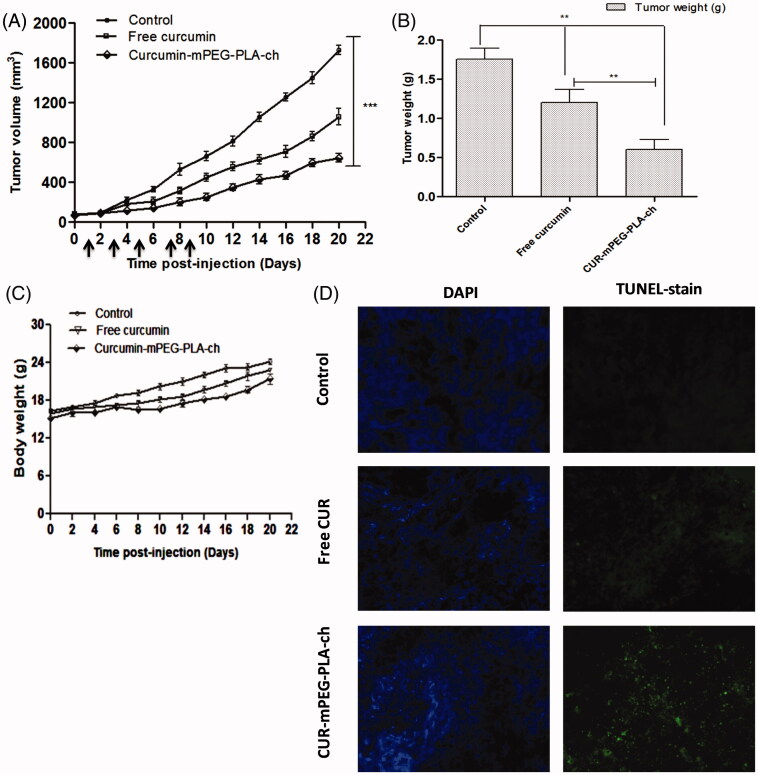
Assessment of *in vivo* therapeutic efficacy of CUR-mPEG–PLA-Ch micelles compared to free CUR administered intraperitoneally in B16F10-tumor-bearing mice. (A and B). A graphical representation of measured tumor volume versus time postinjection, and the weight of the tumor isolated after sacrificing the animal post-treatment; (C) The body weight of mice of different treatment groups plotted against the duration of treatment (D) Apoptosis analysis. Apoptotic cells were detected in frozen tumor sections, determined by TUNEL assay and visualized by fluorescence microscopy. The left panel shows the sections stained with DAPI and the right panel shows the TUNEL staining. Magnifications −20 × objectives.

C57BL/6 mice bearing ∼50–100 mm^3^ of B16F10-xenografted tumors were treated with CUR dose of 25 mg/kg every 2 days for five times. The intraperitoneal injection volume was maintained at ∼200 μL (below 500 μL) to reduce the stress on the animals. Formulations were prepared at CUR concentration of 500 μg/mL to maintain an injection volume of 200 μL and the dose of CUR at 25 mg/kg. The results obtained from the *in vivo* experiments have been represented in Figure 9. The graphical representation of tumor volumes versus time postinjection (over the course of treatment for 22 days) indicated that the treatment with CUR-mPEG–PLA-Ch suppressed the tumor growth at significantly higher rate compared to free CUR-treatment (Figure 9A). Over the 22-day period, the tumor volume increased from 78.4 ± 3.9 to 613.9 ± 11.6 mm^3^ for CUR-mPEG–PLA-Ch, whereas for free CUR, the volume increased from 68.1 ± 5.4 to 917.5 ± 9.7 mm^3^. The tumor weight after isolation was 1.75 ± 0.14, 1.20 ± 0.17 and 0.60 ± 0.13 g for PBS, free CUR and CUR-mPEG–PLA-Ch treatment, respectively (Figure 9B). Further, the treated animals showed no reduction in body weight over the treatment period that indicated that no formulation exhibited marked *in vivo* toxicity (Figure 9C). To determine the extent of apoptosis in the tumor after the treatment, TUNEL assay was performed. Figure 9(D) represented the fluorescence micrograph of tumor tissue sections following TUNEL assay. As visualized under fluorescence microscope, the CUR micelles-treated group showed significantly higher amounts of green dots representative of apoptotic nuclei attributed to FITC-labeled TdT compared to the tumor section from free CUR treatment group. The cell nuclei of tumors treated with PBS exhibited no green fluorescence attributable to FITC-labeled TdT indicating no sign of apoptosis.

## Conclusion

The study has identified a novel polymeric micellar drug delivery system, cholesterol-modified mPEG–PLA micelles, mPEG–PLA-Ch for improved delivery of CUR in cancer. Modification with cholesterol improved the hydrophobicity of the core in mPEG–PLA micelles resulting in superior micellization ability. The newly developed polymeric micelles encapsulated and delivered CUR efficiently in various cancer cell lines *in vitro* and into the tumor *in vivo* that resulted in improved therapeutic efficacy of CUR compared to the treatment with free CUR. The mPEG–PLA-Ch micelles could potentially be utilized to deliver any hydrophobic chemotherapeutic agents, including CUR in cancer. In addition, the study provides a strong rationale for potential utilization of the newly developed CUR-mPEG–PLA-Ch micellar system as a promising anticancer therapy.

## Supplementary Material

supplementary_document.docx

## Notes

**Supplementary material available online.**
