# Supplementary material for: Cholesterol-conjugated poly(D, L-lactide)-based micelles as a nanocarrier system for effective delivery of curcumin in cancer therapy
Source: Drug Deliv. 2017 Feb 3;24(1):209–23. doi: 10.1080/10717544.2016.1245365 (PMC8253141; doi:10.1080/10717544.2016.1245365)
Supplement: supplementary_document.docx [file IDRD_A_1245365_SM7408.docx]

**Cholesterol-conjugated poly(D, L-Lactide)-based micelles as a nanocarrier system for effective delivery of curcumin in cancer therapy**

Preeti Kumari, Omkara Swami Muddineti, Sri Vishnu Kiran Rompicharla, Pratyusha Ghanta, Adithya Karthik B B N, Balaram Ghosh, Swati Biswas*

**Characterization of mPEG-PLA-Ch by IR spectroscopy.**

The infrared spectra of mPEG-PLA (SF-1) shows peak at 1467 and 1343 cm^-1^. The peaks at 2877 cm^-1^ and 1100 cm^-1^ are characteristic of polymer’s methylene group near to oxygen atom and C–O–C stretching of mPEG. Therefore, it is proven that PEG ether linkage exists in the polymer. A strong carbonyl band appears at 1758 cm^-1^ and a C-O stretching band appears at 1187 cm^-1^; which are present in the spectrum of D,L-lactide. In the spectrum of mPEG-PLA-Ch, the band at approximately 1740.7 cm^−1^ was assigned to the stretching vibration of the C=O bond of the carbonate ester, which was different from that of the acyl chloride, at 1779 cm^−1^. The spectrum was consistent with the successful coupling of cholesterol with mPEG-PLA.


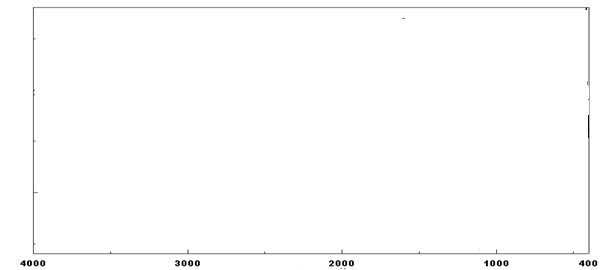


**Transmittance (%)**

**Wavenumber (cm^-1^ )**


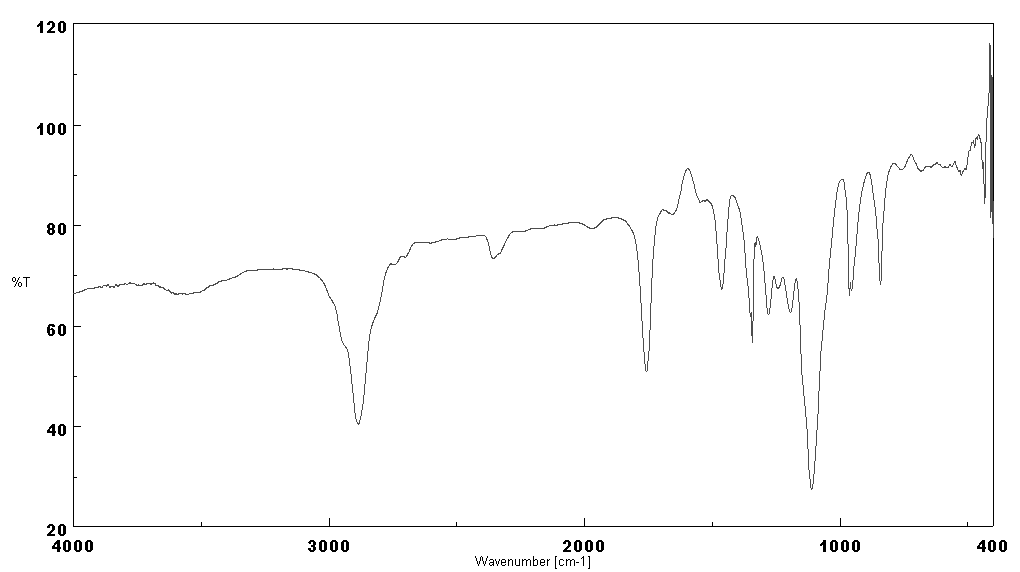

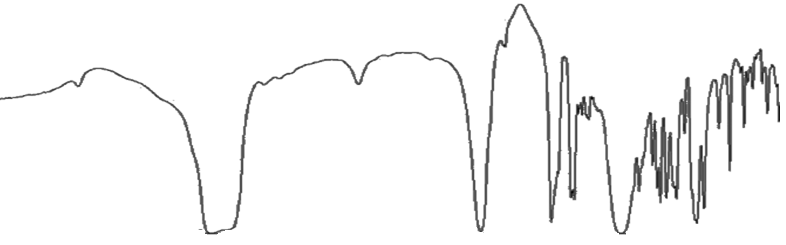

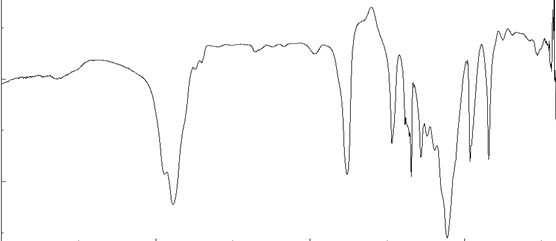


**(a)**

**(b)**

**(c)**

SF- 1: IR spectrum of (a) mPEG-PLA; (b) Cholesteryl chloroformate; (c) mPEG-PLA-Ch
